# Predictive implications of decreased CA19‐9 at 8 weeks during nab‐paclitaxel plus gemcitabine for the induction of second‐line chemotherapy for patients with advanced pancreatic cancer

**DOI:** 10.1002/cnr2.1289

**Published:** 2020-09-23

**Authors:** Kiyotsugu Iede, Terumasa Yamada, Ryo Kato, Masami Ueda, Yujiro Tsuda, Shinsuke Nakashima, Katsuya Ohta, Jin Matsuyama, Masakazu Ikenaga, Shusei Tominaga

**Affiliations:** ^1^ Department of Clinical Oncology Higashiosaka City Medical Center Higashiosaka Japan; ^2^ Department of Gastroenterological Surgery Higashiosaka City Medical Center Higashiosaka Japan

**Keywords:** CA19‐9, gemcitabine, nab‐paclitaxel, pancreatic cancer, S‐1

## Abstract

**Background:**

Second‐line (2L) chemotherapy after nab‐paclitaxel plus gemcitabine (AG) is important for improving the survival of patients with advanced pancreatic cancer (APC). However, many patients fail to receive 2L chemotherapy because of rapid disease progression. Therefore, early recognition of any ineffectiveness during AG might lead to an increased induction rate of 2L chemotherapy.

**Aim:**

We investigated the significance of treatment response at 8 weeks as a predictive factor for the induction of 2L chemotherapy after AG.

**Methods and results:**

From January 2015 to January 2019, 41 patients with APC underwent AG as first‐line chemotherapy at our institute. Thirty‐three patients were evaluated at 8 weeks. Sixteen patients (48%) underwent 2L chemotherapy and 17 (52%) underwent no 2L chemotherapy. Clinical features and treatment response at 8 weeks were, retrospectively, compared among patients. Predictive factors for the induction of 2L chemotherapy were analyzed. Patients with an objective response by 8 weeks received 2L chemotherapy more frequently (*P* = .026). Decreased CA19‐9 (<50%) at 8 weeks was identified as an independent negative predictive factor for the induction of 2L chemotherapy.

**Conclusions:**

Decreased CA19‐9 (<50%) at 8 weeks may indicate the ineffectiveness of AG and signify that a move to 2L chemotherapy may be required without delay.

## 1 INTRODUCTION

Pancreatic cancer (PC) is a highly malignant cancer and 5‐year survival rate was only 8.4% between 2006 and 2010 in Japan.^1^ PC is the fourth leading cause of cancer‐related mortality, and 34 224 people died of PC in Japan in 2017.^2^ Gemcitabine (GEM) has become the standard treatment for advanced PC (APC), showing an improved clinical response and overall survival (OS) compared with fluorouracil (23.8% vs 4.8% and 5.65 vs 4.41 months, respectively).^3^ Since then, GEM‐based combination regimens have been developed^4^, and nab‐paclitaxel plus GEM (AG) has recently demonstrated a survival benefit over GEM alone for patients with metastatic PC in North America, Europe, and Australia (8.5 vs 6.7 months) (MPACT trial).^5^ Second‐line (2L) chemotherapy is important for improved survival.^6^, ^7^, ^8^, ^9^, ^10^ However, the induction rates of 2L chemotherapy between the AG group and the GEM group were 40% and 44%, respectively, and the main reason for the lack of 2L chemotherapy was the rapid progression of PC in the MPACT trial.^7^ In other previous studies, approximately half of patients who received AG or GEM monotherapy as first‐line chemotherapy also failed to move to 2L chemotherapy because of poor performance status (PS) resulting from the disease progression of PC.^6^, ^11^, ^12^ Therefore, early recognition of any ineffectiveness during AG might lead to an increased induction rate of 2L chemotherapy.

Carbohydrate antigen 19‐9 (CA19‐9), a Lewis blood group antigen, is one of the most widely studied tumor markers in patients with advanced PC (APC)^13^, ^14^, ^15^, ^16^ owing to its utility in determining prognosis and response to treatment,^17^, ^18^ and decreased CA19‐9 at 8 weeks was reported as an early predictor of OS in the MPACT trial.^19^ Therefore, in the present study, we retrospectively investigated the significance of CA19‐9 changes at 8 weeks as a predictive factor for the induction of 2L chemotherapy after AG.

## 2 MATERIALS AND METHODS

### 2.1 Patients

From January 2015 to January 2019, 41 patients with APC underwent AG as first‐line (1L) chemotherapy at our institute. Thirty‐three patients were evaluated (eight patients were excluded due to death by 8 weeks (n = 2), CA19‐9 level less than the standard value [n = 4], or CA19‐9 not evaluated at 8 weeks [n = 2]). Of these 33 patients, 16 patients (48%) underwent 2L chemotherapy after AG (2L group) and 17 (52%) underwent no 2L chemotherapy (best supportive care [BSC] group). The patients' demographic information and clinical features at baseline and the treatment response of AG at 8 weeks were retrospectively compared between the 2L and BSC groups. The predictive factors for the induction of 2L chemotherapy were analyzed. This clinical research was approved by the institutional review board (02‐0557‐A). All procedures performed in studies involving human participants were in accordance with the ethical standards of the institutional research committee and with the 1964 Helsinki declaration and its later amendments or comparable ethical standards. Informed consent was obtained from all individual participants included in the study.

### 2.2 Treatment

Beginning in January 2015, AG was administered as 1L chemotherapy for APC until the occurrence of progressive disease or intolerance due to adverse events. After AG, the indications for 2L chemotherapy were based on the patients' general conditions and willingness to receive the treatment. Of 33 evaluable patients at 8 weeks, 16 (48%) underwent 2L chemotherapy: 13 (81%) received S‐1, two (13%) received modified FOLFIRINOX (fluorouracil/leucovorin plus irinotecan plus oxaliplatin), and one (6%) received GEM combined with carbon ion beam therapy. Seventeen patients (52%) received no 2L chemotherapy. The reasons for the lack of 2L chemotherapy were as follows: poor PS because of disease progression in nine patients (53%), severe adverse events associated with AG in three patients (18%) (febrile neutropenia, n = 1; liver dysfunction, n = 1; interstitial pneumonia, n = 1), patient refusal because of severe fatigue caused by AG in four patients (24%), and cholangitis in one patient (6%).

### 2.3 Tumor response

Tumor response was assessed by CT using the Response Evaluation Criteria in Solid Tumors (RECIST version 1.1). Radiological evaluation was undertaken at 8 weeks after the start of AG. The OR was defined as a complete response (CR) or partial response (PR) by 8 weeks. Stable disease (SD) and progressive disease (PD) were defined by RECIST 1.1. CA19‐9 changes were evaluated by comparing baseline levels with those at 8 weeks.

### 2.4 Statistical analysis

Data are expressed as median (range). Statistical analyses were performed using JMP version 13.0 software (SAS Institute, Cary, NC). The differences between the two groups were compared with chi‐squared statistics, Fisher's exact test, and the Mann‐Whitney *U*‐test. OS was estimated using the Kaplan‐Meier method and compared using the log‐rank test, and was calculated from treatment initiation to patient death or censored time. Predictive factors with a *P* value of <.30 in the univariate analysis were entered into a multivariate logistic regression model to determine independent predictors. All *P* values of <.05 were considered significant.

## 3 RESULTS

### 3.1 Patient characteristics at the start of AG

The characteristics of the 33 patients at the start of 1L chemotherapy of AG are summarized in Table 1. Baseline characteristics did not significantly differ between the two groups.

**TABLE 1:** Patients' characteristics at the start of nab‐paclitaxel plus gemcitabine

Factor	2L	BSC	*P* value
	(n = 16)	(n = 17)	
Male:Female	7:9	7:10	.88
Age (years)	71.5 (42‐77)	67 (52‐77)	.69
PS (0‐1:2)	16:0	16:1	.32
Distant metastasis (yes:no)	12:4	14:3	.61
Site of primary tumor (head vs body and tail)	5:11	10:7	.112
Site of metastasis			
Liver	9	10	.88
Lung	5	3	.36
Lymph node	8	10	.61
Peritoneum	5	7	.55
Bone	1	0	.30
No. metastatic sites(≥4 vs 1‐3)	3	1	.26
NLR(>5 vs ≤5)	2:14	4:13	.41
CA19‐9 (median U/ml)	3899	1128	.97

Abbreviations: 2L, second‐line chemotherapy; BSC, best supportive care; CA19‐9, carbohydrate antigen 19‐9; NLR, neutrophil‐to‐lymphocyte ratio; PS, performance status.

### 3.2 CA19‐9 change at 8 weeks

Response to AG at 8 weeks is summarized in Table 2. No patients had a CR according to RECIST by 8 weeks, seven patients had a PR, 11 patients had SD, 13 patients had PD, and two patients were not evaluated. Patients who had a PR were more frequent in the 2L group (*P* = .026) and all seven patients continued AG after 8 weeks. Patients whose CA19‐9 had decreased by at least 25% at 8 weeks were more frequent in the 2L group.

**TABLE 2:** RECIST by 8 weeks and CA19‐9 change at 8 weeks

Factor	2L	BSC	*P* value
	(n = 16)	(n = 17)	
RECIST (PR vs SD/PD/NE) by 8 weeks	6:10	1:16	.026*
CA19‐9 decrease vs increase at 8 weeks	14:2	11:6	.127
CA19‐9 decrease (≥25% vs <25%) at 8 weeks	13:3	7:10	.019*
CA19‐9 decrease (≥50% vs <50%) at 8 weeks	11:5	5:12	.024*
CA19‐9 decrease (≥75% vs <75%) at 8 weeks	7:9	1:16	.011*

Abbreviations: 2L, second‐line chemotherapy; BSC, best supportive care; CA19‐9, carbohydrate antigen 19‐9; NE, not evaluated; PD, progressive disease; PR, partial response; SD, stable disease.

* Statistically significant.

### 3.3 CA19‐9 change at 8 weeks without OR by 8 weeks

The response to AG at 8 weeks without OR by 8 weeks is summarized in Table 3. There was no significant difference in patients with SD or PD according to RECIST by 8 weeks between the 2L and BSC groups. Ten of 11 patients with SD according to RECIST by 8 weeks continued AG after 8 weeks. One of these 11 patients discontinued AG due to patient refusal because of severe fatigue. Six of 13 patients with PD according to RECIST by 8 weeks continued AG according to their doctors' judgement or the patients' willingness after 8 weeks. Seven of those 13 patients discontinued AG at 8 weeks. One of these seven patients discontinued AG because of interstitial pneumonia. CA19‐9 that had decreased by at least 50% at 8 weeks during AG was more frequent in the 2L group. Two patients who were not radiologically evaluated by 8 weeks discontinued AG by 8 weeks due to severe fatigue and febrile neutropenia.

**TABLE 3:** CA19‐9 change at 8 weeks without an objective response by 8 weeks

Factor	2L	BSC	*P* value
	(n = 10)	(n = 16)	
RECIST (SD vs PD) by 8 weeks	5:5	6:8	.73
CA19‐9 decrease vs increase	8:2	10:6	.35
CA19‐9 decrease (≥25% vs <25%) at 8 weeks	7:3	6:10	.107
CA19‐9 decrease (≥50% vs <50%) at 8 weeks	7:3	4:12	.024*
CA19‐9 decrease (≥75% vs <75%) at 8 weeks	4:6	0:16	.006*

Abbreviations: 2L, second‐line chemotherapy; BSC, best supportive care; CA19‐9, carbohydrate antigen 19‐9; PD progressive disease; SD, stable disease.

^*^ Statistically significant.

### 3.4 Univariate and multivariate analyses of predictive factors for the induction of 2L chemotherapy

Univariate and multivariate analyses were performed to analyze predictive factors for the induction of 2L chemotherapy (Table 4). When the univariate analysis was performed for the variables, the significant negative predictive factor for the induction of 2L chemotherapy was decreased CA19‐9 (<50%) at 8 weeks. The multivariate analysis also revealed that decreased CA19‐9 (<50%) at 8 weeks was an independent negative predictive factor for the induction of 2L chemotherapy.

**TABLE 4:** Univariate and multivariate analyses for the induction of 2L chemotherapy

Factor	Univariate		Multivariate	
	HR (95%CI)	*P* value	HR (95%CI)	*P* value
Gender				
Female	1	.88		
Male	1.11 (0.28‐4.42)			
Age (years)				
<65	1	.63		
≥65	0.69 (0.16‐2.97)			
Distant metastasis (yes:no)				
No	1	.61		
Yes	0.64 (0.12‐3.46)			
Site of primary tumor				
Head	1	.117	1	.49
Body and tail	3.14 (0.75‐13.16)		1.80 (0.34‐9.36)	
Liver metastasis				
No	1	.88		
Yes	0.90 (0.23‐3.58)			
Lung metastasis				
No	1	.37		
Yes	2.12 (0.41‐10.9)			
Lymph nodes				
No	1	.61		
Yes	0.70 (0.18‐2.77)			
Peritoneum				
No	1	.55		
Yes	0.65 (0.16‐2.72)			
No. of metastatic sites				
0‐3	1	.28	1	.39
>3	3.69 (0.34‐39.8)		3.25 (0.22‐48.09)	
NLR				
≤5	1	.42		
>5	0.46 (0.07‐2.98)			
CA19‐9 (U/ml)				
<72 × ULN	1	.60		
≥72 × ULN	1.45 (0.37‐5.70)			
CA19‐9 decrease				
<50%	1	.028*	1	.047*
≥50%	5.28 (1.20‐23.3)		4.94 (1.02‐24.00)	

Abbreviations: 2L, second‐line; CA19‐9, carbohydrate antigen 19‐9; CI, confidence interval; HR, hazard ratio; NLR, neutrophil‐to‐lymphocyte ratio.

^*^ Statistically significant.

## 4 DISCUSSION

To reveal predictive factors for the induction of 2L chemotherapy during AG, we retrospectively studied the clinical features of patients at our institute. Patients with an OR by 8 weeks received 2L chemotherapy more frequently than patients without an OR by 8 weeks. Decreased CA19‐9 (<50%) at 8 weeks was an independent negative predictive factor for the induction of 2L chemotherapy in the multivariate analysis.

The present study is the first to reveal the significance of RECIST evaluation and decreased CA19‐9 at 8 weeks during AG as predictive markers for the induction of 2L chemotherapy for patients with APC. In the MPACT trial, a higher percentage of patients who received 2L chemotherapy had a Karnofsky PS (KPS) of 90‐100 at baseline, and KPS, CA19‐9, and NLR at the end of AG were reported as surrogates for the administration of subsequent therapy.^7^ In our previous study, more patients with a good PS (0‐1 vs ≥2) at the end of AG or a long progression‐free survival time for AG (≥3.9 vs <3.9 months) received S‐1 as 2L chemotherapy after AG.^6^ However, an early predictive marker during AG for the induction of 2L chemotherapy after AG remained unknown. Patients who exhibited an OR by 8 weeks during AG would be recommended to continue AG after 8 weeks. Among patients without an OR by 8 weeks, disease control can be objectively evaluated by CA19‐9 change at 8 weeks and can predict that patients who had decreased CA19‐9 (<50%) at 8 weeks were likely to lose the opportunity to move to 2L chemotherapy. Therefore, we might consider performing CT by 8 weeks and then again by 16 weeks but suggest that an additional CT scan should be performed by 12 weeks for those patients with a poor response to AG. We should also pay greater attention to the general condition of patients to recognize disease progression and to decide upon a move to 2L chemotherapy without delay for those patients with a poor response to AG. However, the failure of disease control by AG may indicate an aggressive malignancy, potentially resulting in a poor prognosis in patients with decreased CA19‐9 (<50%). Therefore, it remains unclear whether the induction of 2L chemotherapy without delay for patients with decreased CA19‐9 (<50%) at 8 weeks during AG really contributes to prolonged OS. A prospective study is urgently required to validate the real efficacy of induction of 2L chemotherapy for these patients.

The main reason for the lack of 2L chemotherapy was poor PS due to disease progression, followed by patient refusal due to severe fatigue. Because this severe fatigue might be associated with poor PS due to disease progression, as well as adverse events, the evaluation of treatment response as a predictive marker for the induction of 2L chemotherapy might be useful for these patients as well as for patients with a poor PS resulting from disease progression. However, because the lack of 2L chemotherapy was also due to poor PS resulting from severe adverse events and cholangitis, the evaluation of treatment response might not be useful for these patients. Moreover, the evaluation of CA19‐9 changes was not available to patients who were negative for the Lewis antigen. This is because the measured level of CA19‐9 becomes a false‐negative despite the high duke pancreatic monoclonal antigen type 2 (DUPAN‐2) levels in these patients.^20^, ^21^, ^22^ Further investigations are needed to identify a predictive marker for the induction of 2L chemotherapy among patients with poor PS resulting from severe adverse events or cholangitis and who are negative for the Lewis antigen.

This study has several limitations. We were unable to eliminate any potential selection bias because this was a single‐center retrospective study and the statistical power was limited by the small sample size. According to the RECIST criteria, patients who experienced PD by 8 weeks would be recommended to end AG and consider other agents. However, in real clinical practice, we sometimes encounter patients in whom it is difficult to distinguish SD from PD according to RECIST by 8 weeks and in whom there was no obvious evidence of an actionable drug in 2L chemotherapy after AG, despite S‐1 being administered in 2L chemotherapy after AG as a standard‐of‐care in Japan.^6^ Therefore, 6 of 13 patients with PD according to RECIST by 8 weeks continued AG. Considering these patients, we investigated predictive factors for the induction of 2L chemotherapy among all evaluated patients.

In conclusion, decreased CA19‐9 (<50%) at 8 weeks may indicate the ineffectiveness of AG and suggest that an immediate move to 2L chemotherapy is required.

## CONFLICT OF INTEREST

The authors declare no conflicts of interest.

## AUTHORS' CONTRIBUTION

All authors had full access to the data in the study and take responsibility for the integrity of the data and the accuracy of the data analysis. *Conceptualization*, Kiyotsugu Iede; *Methodology*, Kiyotsugu Iede; *Investigation*, Kiyotsugu Iede, Yujiro Tsuda, and Shinsuke Nakashima; *Formal analysis*, Kiyotsugu Iede and Terumasa Yamada; *Resources*, Kiyotsugu Iede, Ryo Kato, Masami Ueda, Yujiro Tsuda, Shinsuke Nakashima, Katsuya Ohta, Jin Matsuyama, and Masakazu Ikenaga; *Writing‐original draft*, Kiyotsugu Iede; *Writing‐review & editing*, Kiyotsugu Iede; *Visualization*, Kiyotsugu Iede; *Supervision*, Terumasa Yamada and Shusei Tominaga.

## ETHICS STATEMENT

All procedures performed in studies involving human participants were in accordance with the ethical standards of the institutional research committee and with the 1964 Helsinki declaration and its later amendments or comparable ethical standards.

## INFORMED CONSENT

Informed consent was obtained from all individual participants included in the study.

## Data Availability

The data that support the findings of this study are available on request from the corresponding author. The data are not publicly available because of privacy or ethical restrictions.
